# Measurements From Ears With Endolymphatic Hydrops and 2-Hydroxypropyl-Beta-Cyclodextrin Provide Evidence That Loudness Recruitment Can Have a Cochlear Origin

**DOI:** 10.3389/fsurg.2021.687490

**Published:** 2021-10-05

**Authors:** Shannon M. Lefler, Robert K. Duncan, Shawn S. Goodman, John J. Guinan, Jeffery T. Lichtenhan

**Affiliations:** ^1^Department of Otolaryngology, Washington University School of Medicine in St. Louis, Saint Louis, MO, United States; ^2^Department of Otolaryngology-Head and Neck Surgery, Kresge Hearing Research Institute, University of Michigan, Ann Arbor, MI, United States; ^3^Department of Communication Sciences and Disorders, University of Iowa, Iowa City, IA, United States; ^4^Eaton-Peabody Laboratories, Massachusetts Eye and Ear, Boston, MA, United States; ^5^Department of Otolaryngology, Harvard Medical School, Boston, MA, United States

**Keywords:** endolymphatic hydrops, low-frequency hearing, loudness recruitment, auditory nerve overlapped waveform, 2-hydroxypropyl-beta-cyclodextrin

## Abstract

**Background:** Loudness recruitment is commonly experienced by patients with putative endolymphatic hydrops. Loudness recruitment is abnormal loudness growth with high-level sounds being perceived as having normal loudness even though hearing thresholds are elevated. The traditional interpretation of recruitment is that cochlear amplification has been reduced. Since the cochlear amplifier acts primarily at low sound levels, an ear with elevated thresholds from reduced cochlear amplification can have normal processing at high sound levels. In humans, recruitment can be studied using perceptual loudness but in animals physiological measurements are used. Recruitment in animal *auditory-nerve* responses has never been unequivocally demonstrated because the animals used had damage to sensory and neural cells, not solely a reduction of cochlear amplification. Investigators have thus looked for, and found, evidence of recruitment in the auditory central nervous system (CNS). While studies on CNS recruitment are informative, they cannot rule out the traditional interpretation of recruitment originating in the cochlea.

**Design:** We used techniques that could assess hearing function throughout entire frequency- and dynamic-range of hearing. Measurements were made from two animal models: guinea-pig ears with endolymphatic-sac-ablation surgery to produce endolymphatic hydrops, and naïve guinea-pig ears with cochlear perfusions of 13 mM 2-Hydroxypropyl-Beta-Cyclodextrin (HPBCD) in artificial perilymph. Endolymphatic sac ablation caused low-frequency loss. Animals treated with HPBCD had hearing loss at all frequencies. None of these animals had loss of hair cells or synapses on auditory nerve fibers.

**Results:** In ears with endolymphatic hydrops and those perfused with HPBCD, auditory-nerve based measurements at low frequencies showed recruitment compared to controls. Recruitment was not found at high frequencies (> 4 kHz) where hearing thresholds were normal in ears with endolymphatic hydrops and elevated in ears treated with HPBCD.

**Conclusions:** We found compelling evidence of recruitment in auditory-nerve data. Such clear evidence has never been shown before. Our findings suggest that, in patients suspected of having endolymphatic hydrops, loudness recruitment may be a good indication that the associated low-frequency hearing loss originates from a reduction of cochlear amplification, and that measurements of recruitment could be used in differential diagnosis and treatment monitoring of Ménière's disease.

## Introduction

Dynamic range alterations accompany disorders of many sensory systems including the auditory system. The dynamic range of hearing is altered when trauma or disease elevates the thresholds of low-level sounds, but the loudness of high-level sounds is within normal limits. An elevated threshold with an abnormally fast growth of loudness that achieves normal loudness at high levels is called “recruitment” [e.g., (1–3)]. Recruitment demonstrates that audiometric sensorineural hearing loss is more complex than a simple linear reduction of sound, such as from disorders of the middle ear or the use of hearing-protective devices (e.g., ear muffs or earplugs). In animal models, recruitment is typically studied with measurements of physiologic responses (e.g., auditory nerve compound action potentials -CAPs), not by perceptual loudness. Here we use the term “response recruitment” to distinguish recruitment measured by a physiologic response from “loudness recruitment” which is recruitment measured by a subjective or psychophysical method.

The traditional proposed pathophysiologic mechanism of recruitment is that cochlear amplification has been reduced. Cochlear amplification is a process that increases basilar-membrane (BM) vibrations by mechanisms that saturate at high levels. Cochlear amplification improves sensitivity to low-level sounds, but does not appear to play an important role in responses to high-level sounds. Cochlear amplification requires outer-hair-cell (OHC) electromotility, and functional impairment or loss of OHCs produces hearing threshold elevation (i.e., hearing loss) that can be many tens of dB. In contrast to low-level responses, most auditory responses to high-level sounds are unaffected by reductions of cochlear amplification. Thus, a reduction of cochlear amplification results in hearing threshold elevation but normal responses to high-level sounds with a resulting higher rate of response growth (i.e., in recruitment).

The traditional interpretation of recruitment originated from measurements of BM motion. Reductions in cochlear amplification, such as from damage or stimulation of olivocochlear efferents, change the growth of BM motion with sound level, with vibration threshold being elevated but the motion remaining within normal limits for high-level sounds [e.g., (4, 5)]. Following reductions of cochlear amplification, measurements of both loudness and BM motion show increased thresholds, increased growth with sound level and normal amplitudes at high levels. *The striking similarity between these is the main source of the traditional interpretation that recruitment has a cochlear origin* [e.g., (6, 7)].

There have been no published data showing clear response recruitment in measurements from the auditory nerve of damaged or diseased ears in which the damage was restricted to a reduction of cochlear amplification (8, 9). The lack of auditory-nerve data showing recruitment may be a consequence of the lack of animal models with reduced cochlear amplification but preserved synaptic activity between inner hair cells (IHCs) and auditory nerve fibers. For example, cochleae that have been treated with salicylate or acoustic overexposure can have reduced cochlear amplification, but can also have impairment of IHCs and the IHC synapses with auditory nerve fibers [e.g., (10, 11)]. The lack of data showing unequivocal recruitment in auditory nerve measurements has led investigators to reject the traditional interpretation that loudness recruitment originates in the cochlea and to consider alternative origins such as that loudness recruitment originates in the auditory central nervous system (CNS) [e.g., (8)].

We hypothesized that a pure reduction of cochlear amplification, without damage to IHCs, auditory nerve fibers, or endocochlear potential, would show recruitment that arises in the cochlea and is manifested in auditory-nerve responses. Here we show recruitment in auditory-nerve measurements from two guinea pig models: (1) guinea-pig ears that underwent surgery to ablate the endolymphatic sac, a procedure that induces endolymphatic hydrops that can be seen histologically at 30 postoperative days and that causes low-frequency hearing threshold elevation soon after the ablation (12–14), and (2) naïve (i.e., never operated on) animals that underwent cochlear perfusions of 13 mM 2-Hydroxypropyl-Beta-Cyclodextrin (HPBCD) in artificial perilymph. HPBCD can be used to treat Niemann-Pick type C disease, Alzheimer's disease, and atherosclerosis, but causes hearing loss [(15) p. 1,017]. Ears with endolymphatic hydrops and animals that underwent acute cochlear perfusion with low-dose HPBCD did not have loss of cochlear hair cells or afferent auditory nerve synapses, and the hearing loss appears to arise from a reduction of cochlear amplification (14, 16). Ears with endolymphatic hydrops as well as those treated acutely with HPBCD are thus well suited to address the question of whether the traditional interpretation of recruitment is correct. Moreover, if the degree of response recruitment correlates with the severity of endolymphatic hydrops, it is possible that clinical measurements of loudness recruitment can be used as a functional assessment of the presence of endolymphatic hydrops in patients with Ménière's disease.

## Methods

### Overview

**Figure 1:**
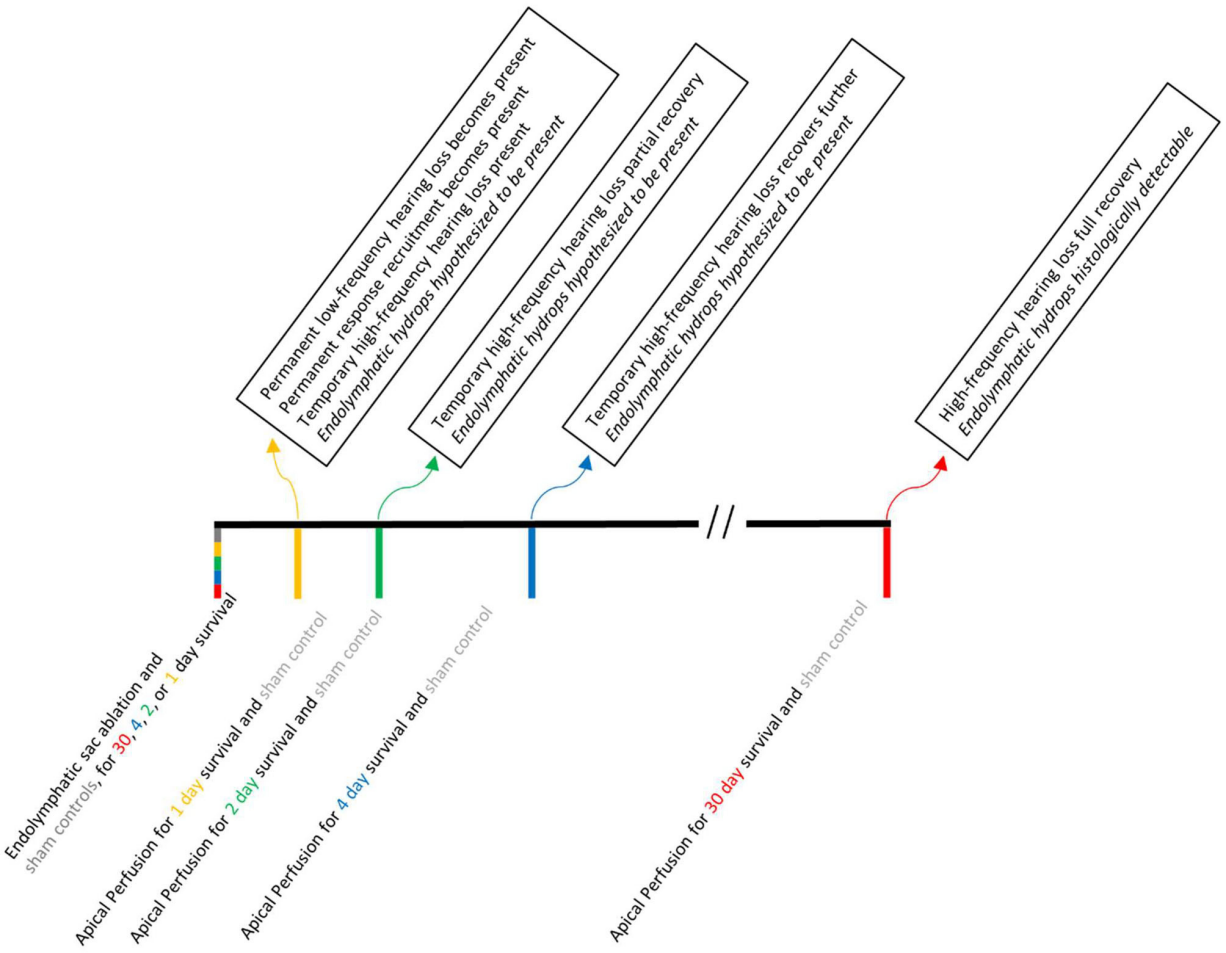
A time line of the experimental manipulations (below the line) and results (above the line) associated with endolymphatic sac ablation. Acute apical perfusion of HPBCD in otherwise naïve ears are not shown because all of the experimental manipulations and testing were done on the same day.

The right ears of NIH-strain pigmented guinea pigs of either sex weighing at least 400 g were used. Animals were assigned to one of two groups: an endolymphatic-sac-surgery group or an HPBCD group. We provided the full details of the endolymphatic sac surgery in a video-based publication (14). The key steps are to visualize the extra-osseous portion of the endolymphatic sac, to use fine picks to destroy the intraosseous portion of the endolymphatic sac, and to fully disarticulate it from the extra-osseous portion. Middle ear structure was not disrupted during the endolymphatic sac ablation surgery. The endolymphatic sac group had 43 guinea pigs, with two animals used here for the first time and 38 used previously in Lee et al. (12) or Valenzuela et al. (13, 14). The eight control animals for the endolymphatic sac group (four sham surgery and four naïve) were also used in Lee et al. (12) and Valenzuela et al. (13, 14). The sham surgery involved visualizing the endolymphatic duct though not ablating it. Results from sham-surgery and naïve animals were found to be similar (12). Operated animals underwent a second surgery to make cochlear function measurements at pre-defined postoperative time points: 1, 2, 4, or 30 days (Figure 1). The number of animals used in each figure is provided in its legend.

Three guinea pigs received cochlear perfusions of HPBCD. The surgery for the HPBCD animals was nearly identical to the surgery to make cochlear function measurements in the endolymphatic sac animals, with one additional step to directly administer HPBCD in artificial endolymph using the apex-to-base cochlear perfusion technique that is routinely used in our lab (16–21). Control measurements for the HPBCD group were made from each ear before perfusion.

Cochlear histology was done on the animals with endolymphatyic sac ablation (both ears), ears with HPBCD perfusions, and selected ears from control animals. The histological procedure is fully described in Lee et al. (12) where it was used to assess endolymphatic hydrops and in Lichtenhan et al. (16) where it was used to assess structural integrity following HPBCD. The histological assessment did not reveal any sensory cell loss and the immunohistoflurescence-based confocal microscopy did not show any cochlear synapse loss following endolymphatic sac ablation. At present, we do not know the origin of the low-frequency hearing loss associated with endolymphatic hydrops in guinea pigs, although we speculate that it is due to endolymphatic hydrops that does not show up in histology (12). This animal model is consistent with findings from human temporal bone studies of patients with Ménière's disease (22).

### Sound Calibration

During the experiments, a hollow ear bar (5, 0.322 cm i.d.) was attached to the bony portion of the right ear canal using a stereotaxic device. One end of the bar was wrapped in a portion of the cut ear canal soft tissue to form a sound delivery port with no acoustic leaks. Stimuli were presented via an ER-10X (Etymotic Research) probe connected to the other end of the hollow ear bar. Prior to the experiments, the sound delivery system was calibrated using a 1/8” GRAS type 40P reference microphone and custom-made coupling device. To calibrate system output, the hollow ear bar was coupled to one end of a cavity having dimensions and volume similar to the bony portion of the ear canal, and the reference microphone was coupled to the opposite end. Stimuli were played through the ER-10X probe loudspeakers into the ear bar and measured by the reference microphone. A transfer function for the sound source to sound pressure at the plane of the tympanic membrane was obtained and applied to all stimuli used in this experiment.

The ER-10X probe microphone was used for recording otoacoustic emissions, not for setting stimulus levels. The ER-10X probe microphone was calibrated using a copper tube (1.83 m; 0.635 cm i.d.) that was closed at one end and had a sound source placed in the opposite end. The ER-10X probe and a reference microphone (1/8” GRAS type 40P) were sealed in small holes located ~2.5 cm from the sound source. The probe and reference microphones were located opposite to each other and perpendicular to the long axis of the tube. The microphone inlet (probe) and diaphragm (reference) were flush with the wall of the tube. A train of click stimuli was played through the sound source, and the incident wave was measured simultaneously by the probe and reference microphones. Clicks were spaced in time to allow internal reflections in the tube to decay into the noise floor before the next click in the train was presented. Measurements were averaged and temporally windowed to include the incident wave only. A transfer function of the ER-10X probe microphone relative to the reference microphone was created and used to achieve a flat probe microphone response from 0.1 to 34 kHz.

### Physiologic Measurements

Animals were sedated with an intraperitoneal injection of 100 mg/kg Inactin hydrate (i.e., thiobutabarbital sodium), after which the head and neck areas were shaved. A tracheotomy was done to artificially ventilate and sustain anesthesia with ~1.2% of isoflurane in oxygen gas. Ventelation volume was adjusted to maintain 5% end-tidal CO2. A pulse oximeter (Capno True Amp, Bluepoint Medical) was used to monitor O2 saturation, expired CO2 level, and heart rate. A DC-powered heating blanket and rectal thermometer system (Homeothermic Blanket with Flexible Probe from Harvard Apparatus) monitored and maintained body temperature at 38°C. The double-walled sound attenuated room where physiologic measurements were made was heated so that the area immediately around the animal was ~25°C. The guinea pig's head was secured with a bite bar, snout clamp, a hollow ear bar on the right side, and solid ear bar on the left side. When in the supine position, a canula was inserted into the left jugular vein and Lactated Ringer solution (0.5 mL/h) was administered to maintain hydration. The right bulla was accessed ventrally by removing soft tissue and the jaw.

Cochlear function measurements were made using Auditory Research Lab audio software (ARLas, https://github.com/myKungFu). A computer running custom MATLAB software (The MathWorks, Inc., Natick, MA) and the software utility Playrec (23) was used. Stimuli were generated in MATLAB, digitized at 96 kHz, presented to a 24-bit sound card (RME Fireface 802) and routed to an acoustic probe system (ER-10X) that was coupled to the hollow ear bar. Otoacoustic emission (OAE) measurements were made using the acoustic probe system connected to the sound card. Once the ear bars were in place and the head secured, the bulla was opened slightly and OAE measurements were made. The round-window-niche electrode was positioned. Vecuronium bromide (0.1 mg / kg) was administered through the jugular-vein cannula to prevent middle ear muscle contractions. Auditory Nerve Overlapped Waveform (ANOW) and auditory nerve compound action potentials (CAP) measurements were made (details below). These round-window-electrode voltage measurements were band pass filtered at 0.1–3 kHz and amplified 10,000 times (GRAS CP511, Astro-Med, Inc.).

### Otoacoustic Emission Measurements

Distortion product otoacoustic emissions (DPOAEs) at 2f1-f2 were measured with paired primary tones that had durations of 1 s, frequencies f2/f1 = 1.22 and levels L1 & L2 of 60 and 50 dB SPL, respectively. f2 frequencies ranged from 1 to 30 kHz in 2 kHz steps, with 12 repetitions presented at each step. Noise floor measurements were made using the standard error of the DPOAE amplitude converted to dB SPL.

Stimulus frequency otoacoustic emission (SFOAEs) were evoked with probe tones having 250 ms duration, 10 ms rise/fall, and 40 dB SPL sound level. The double-evoked extraction method was used, with a suppressor tone presented 50 Hz above the SFOAE frequency at a level of 60 dB SPL (24–26). The double-evoked method eliminates the stimulus along with system distortion, leaving the otoacoustic emission. Probe tones ranged from 1 to 30 kHz in 2 kHz steps and were presented with 24 repetitions. Noise floor measures were estimated using the standard error of the mean of the SFOAE measurements converted to dB SPL.

### Measurements of Auditory-Nerve Responses

Measurements of auditory-nerve responses were made with an Ag/AgCl ball electrode placed in the round-window niche (non-inverting) and platinum needle electrodes placed in the exposed musculature of the right jaw (inverting) and neck (ground).

### ANOW Measurements and Calculations

ANOW measurements were made with tone bursts (33.3 ms duration) presented in alternating polarity (92 repetitions) at 300, 480, 720, and 1,020 Hz. In this report, these frequencies will be referred to as 300, 500, 700, and 1,000 Hz, respectively. The cochlear microphonic follows the sinusoidal stimuli, but neural excitation occurs mostly during one phase of the tone, for low-frequency tones of low- to moderate-levels. Averaging the response of alternating tone bursts cancels the cochlear microphonic and overlaps the phase-locked neural firing. The ANOW measurements used only the second harmonic of the overlapped response, which selects the auditory-nerve response (27). Recorded ANOW waveforms were bandpass filtered using an FIR filter (600–1,600 Hz passband). Filtered waveforms were corrected for filter group delay and checked for high-amplitude artifacts using a quartile-based detection method (28). The temporal locations of artifacts were recorded, but the artifacts were not removed. Weighted least-squares fits were used to determine the coefficients associated with sinusoids of twice the probe frequency in cosine and (minus) sine phase. The weights on each waveform sample were set to 1 (no artifact) or 0 (artifact present). This method removed the effects of short, infrequent artifacts while preserving the rest of the information in the waveforms, as well as avoiding splatter from edge discontinuities. ANOW waveforms were fit individually, and the resulting coefficients were stored as vectors in complex rectangular form (in a manner similar to complex Fourier coefficients, but at a single frequency). The mean of the stored coefficients was taken as the signal, and the standard error of the mean was taken as the noise floor. ANOW magnitude and phase were computed in the usual way as the square root of the sum of the squares of the real and imaginary parts, and as the four-quadrant arctangent of the ratio of imaginary and real parts.

### CAP Measurements and Calculations

CAP measurements were made in response to tone bursts with 13.9 ms durations (1.0 ms rise/fall) presented with 128 repetitions of alternating polarity. Tone burst presentations were interleaved with periods of silence (13.9 ms duration), so that each alternating-polarity pair was presented at a repetition rate of 14.38/s. The sound level of the tone bursts were varied from 10 to 80 dB SPL in 5 dB steps.

Recorded CAP waveforms were bandpass filtered using an FIR filter (150–1,500 Hz passband). Filtered waveforms were corrected for filter group delay and checked for high-amplitude artifacts using a quartile-based detection method. Waveforms containing artifacts were removed from subsequent analysis. An automated peak-picking algorithm identified the amplitudes and latencies of N1 and P1 for each waveform. Results from the automated algorithm accurately reflected the investigators visual assessments of the amplitudes and latencies.

### Curve Fitting to Response-Amplitude-vs.-Sound-Level Functions

CAP recordings were obtained in 5 dB steps from 10 to 80 dB SPL. For each test frequency, this resulted in 14 peak-to-peak amplitude and peak-delay values. In order to characterize the growth of these values as a function of stimulus level, amplitude and delay were fit (separately) with weighted smoothing splines. The smoothing coefficient (0.005) was determined empirically by examining many data sets and choosing a single value that, across animals, yielded an appropriate amount of smoothing while still retaining essential features of the level series. The weighting values were the signal-to-noise ratio at each stimulus level. The weighting values were set to zero for responses with signal-to-noise ratios of < 6 dB. The spline fit was interpolated to yield values with 1-dB spacing, and these densely-spaced spline curves were differentiated to give growth rates. A similar process was used to find the growth rates of ANOW responses, except that the RMS magnitude (in dB) of the response was used instead of peak-to-peak amplitude, and the phase delay of the response was used instead of the peak delays of the CAP waveform. Our slope measure of “response recruitment” was calculated as the median of the derivative values at sound levels at and above threshold for each frequency of each ear.

## Results

### Recruitment in Ears With Endolymphatic Hydrops

**Figure 2:**
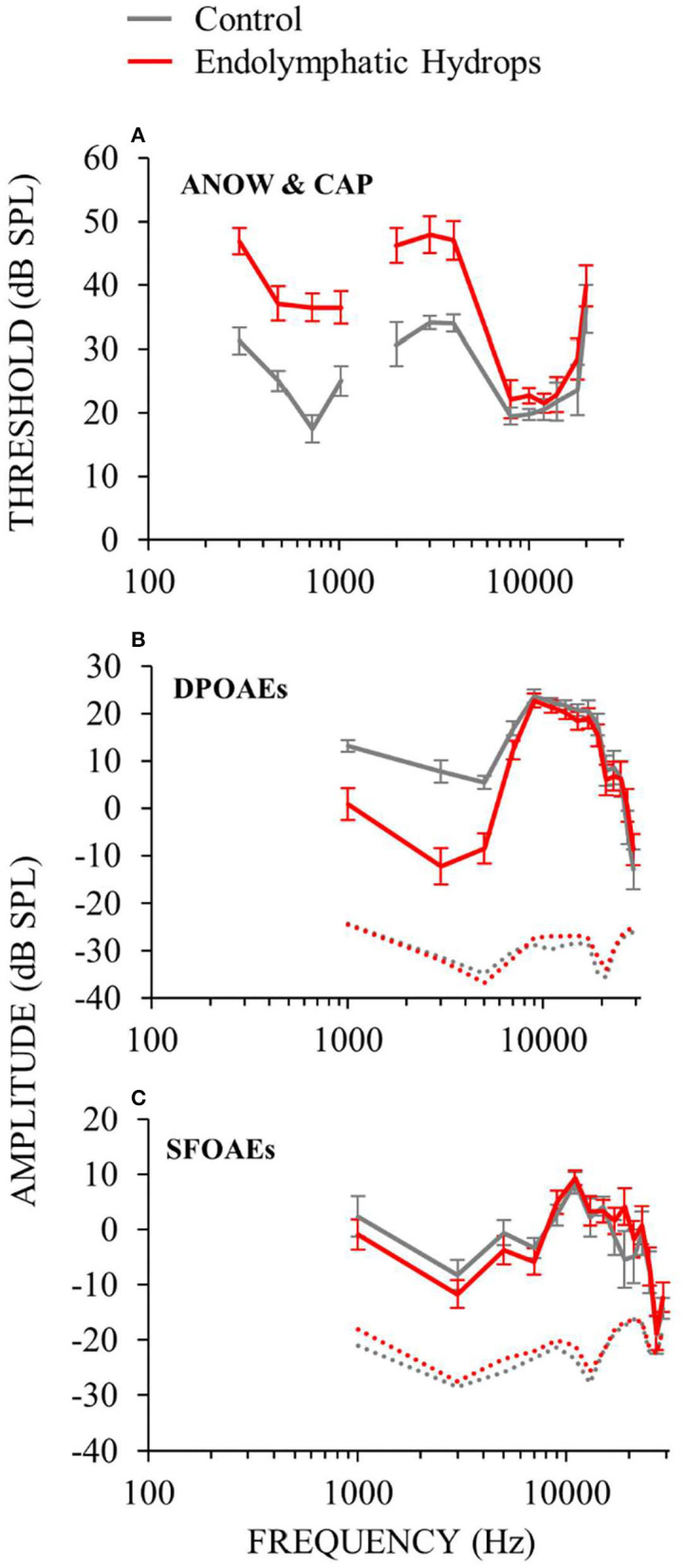
Cochlear function measurements from experimental (red) and control (gray) animals. Data from the right ears of 18 guinea pigs 30 days after ablation of the endolymphatic sac, and from 8 control animals, 4 of which had undergone sham surgery. Error bars are one standard error of the mean. Operated animals had hearing dysfunction at low (≤ 1) and mid (2–4 kHz) frequencies, but not high frequencies. See elevated hearing thresholds **(A)**, decreased DPOAEs **(B)** and decreased SFOAEs **(C)**.

Cochlear function measurements from animals 30 days after endolymphatic sac ablation that induced endolymphatic hydrops are shown in Figure 2. Auditory neural threshold measurements were made with ANOWs for low frequencies (≤ 1 kHz) and with CAPs for mid (2–4 kHz) and high frequencies (8–20 kHz; Figure 2A). Thresholds were elevated relative to control for low- and mid-frequencies and were within normal limits for high-frequencies (Figure 2A). This configuration is similar to that found in human patients with Ménière's disease (22). OAE measurements were made only at frequencies ≥ 1 kHz, due to poor signal-to-noise ratios at lower frequencies. DPOAE amplitudes were decreased relative to controls for mid-frequencies (1–4 kHz), and were within normal limits for other frequencies (Figure 2B). SFOAE amplitudes did not differ significantly from controls (Figure 2C). These OAE results are similar to those we have reported previously (12, 14). Overall, ablation of the endolymphatic sac and the resulting endolymphatic hydrops were associated with low- and mid-frequency hearing dysfunction.

**Figure 3:**
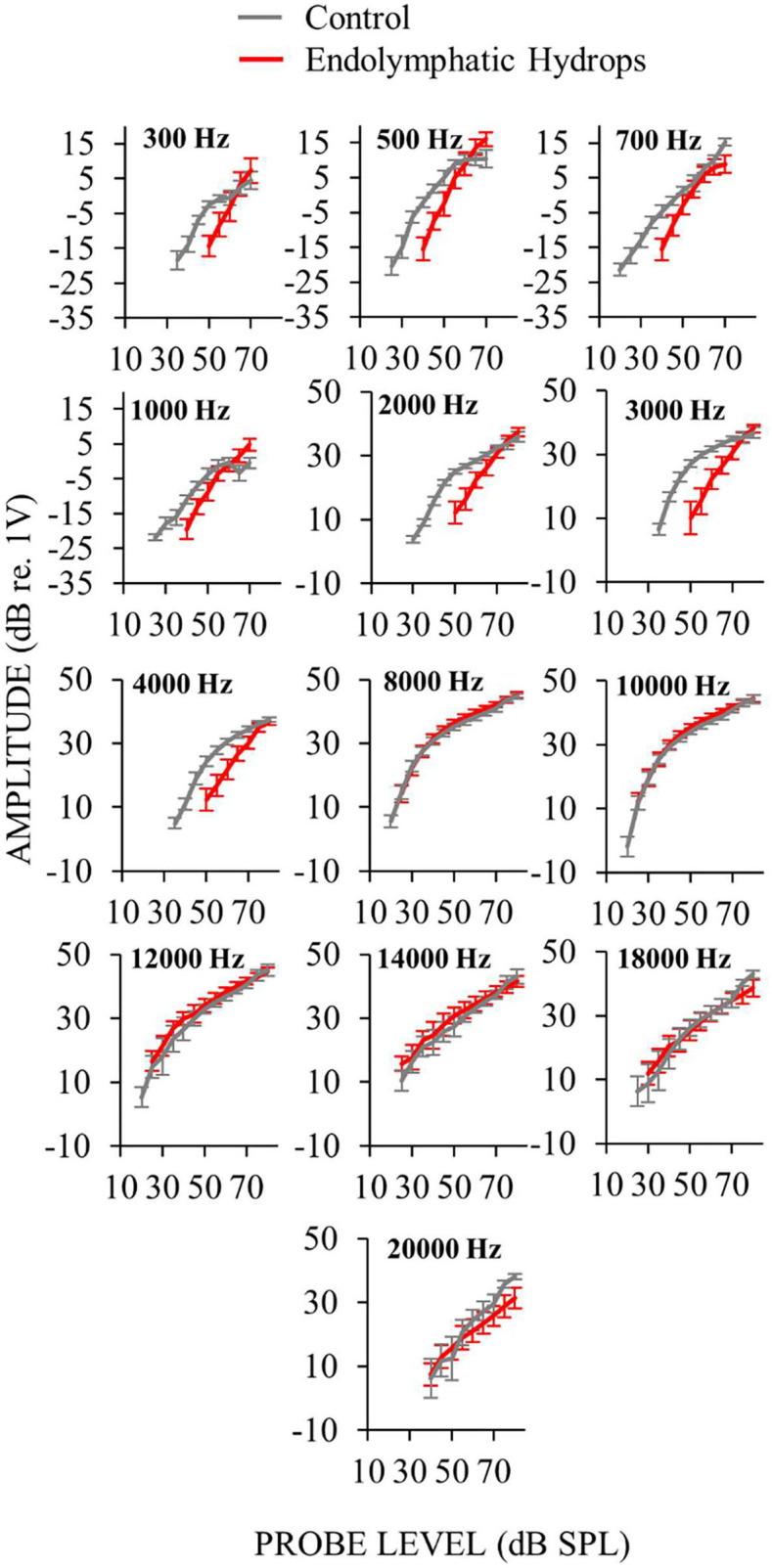
Auditory-nerve response amplitudes from ears with endolymphatic sac ablation (red) and control ears (gray) as functions of sound level for many frequencies. Measurements at low-frequencies (≤ 1 kHz) were made with ANOW and at mid- and high-frequencies (2–4 and ≥ 8 kHz) were made with CAPs. Only measurements that were at, and above, threshold are plotted. Control animals were naïve or underwent a sham-surgery. Points are averages from 18 (low frequencies) or 15 (mid and high frequencies) ears with endolymphatic sac ablation, and eight control ears. Error bars are one standard error of the mean. Response recruitment was seen at low and mid frequencies in that thresholds were elevated but responses converged to near normal at high-levels.

Auditory-nerve responses (ANOW at low frequencies, and CAPs at mid and high frequencies) from ears with endolymphatic sac ablation and from control animals, were measured over a wide range of sound levels (Figure 3). This enabled the assessment of cochlear neural response recruitment throughout the frequency range of hearing. *Ears with endolymphatic sac ablation showed response recruitment at low and mid frequencies in that thresholds were elevated, response amplitude grew faster than normal with level, and supra-threshold responses were within the normal range*. At high sound levels, response amplitudes for low and mid frequencies in ears with endolymphatic sac ablation were occasionally hyper-responsive compared to control (i.e., 0.3, 0.5, and 1 kHz in Figure 3). At high frequencies, CAP amplitudes from ears with endolymphatic sac ablation were essentially indistinguishable from control ears, except that responses at 20 kHz were reduced compared to controls at the highest stimulus levels.

**Figure 4:**
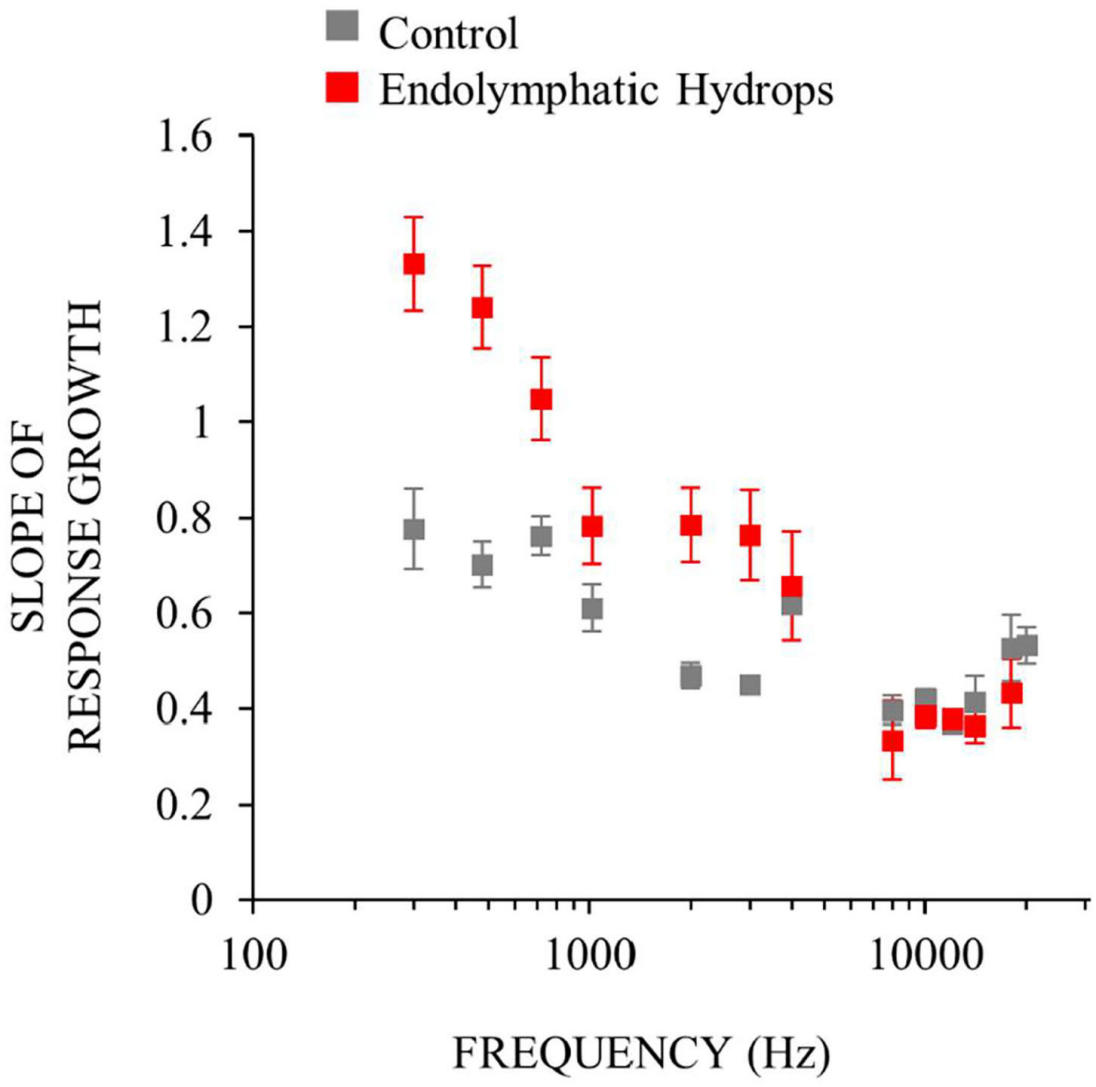
Neural response recruitment quantified by the median slopes of response-amplitude vs. sound-level functions, vs. frequency. Points are medians. Error bars are one standard error of the mean. Data from ears with endolymphatic sac ablation are red, and from control ears are gray. Data are from the same measurements as Figure 3.

To measure response-growth (slopes), the auditory-nerve response-amplitude vs. sound-level functions of individual ears were fit with smoothing splines, and the spline slopes were calculated. Although the slopes varied with sound level, compact estimates of the overall growth rates were obtained by taking the *median* slope of each function. This slope metric allowed comparison of rates of growth across multiple frequencies and animals. For each frequency, Figure 4 shows the median across ears of the median slopes. The median slope values for ears with endolymphatic sac ablation (red in Figure 4) were higher than controls (gray) for low (≤ 1 kHz) and mid (2–4 kHz) frequencies, and were little different than control ears for high (≥ 8 kHz) frequencies (Figure 4). The steeper slopes in the ears with endolymphatic sac ablation are consistent with these ears having response recruitment at low and mid frequencies. In contrast, the lower slopes of the control ears are consistent with the more compressive growth produced by cochlear amplification.

**Figure 5:**
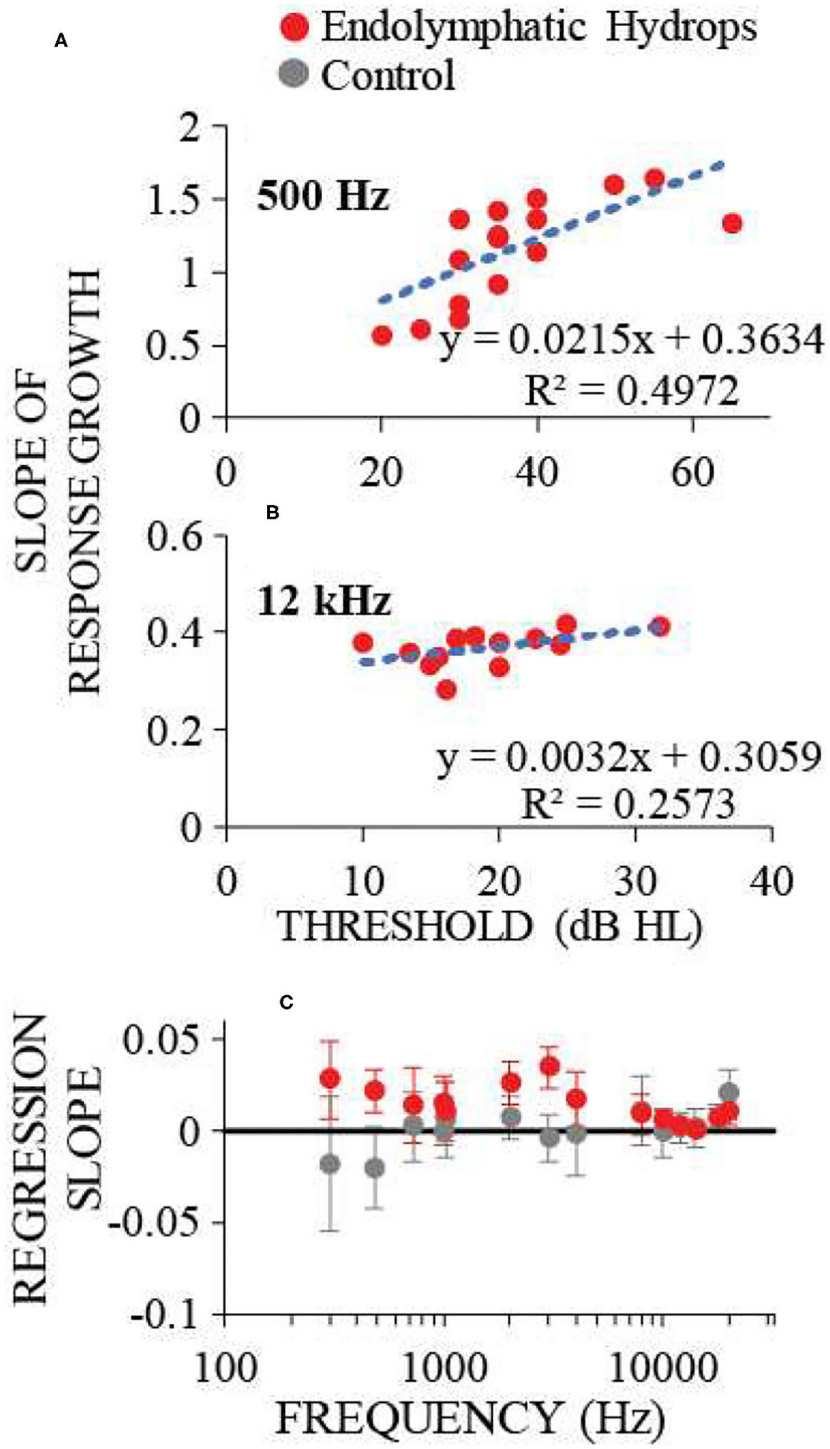
The dependence on hearing threshold of the slope metric of response recruitment. **(A,B)** show the slope metric for response recruitment vs. the threshold of that ear at the test frequency, for two example frequencies with one point for each ear operated on to remove the endolymphatic sac. Dotted lines are linear regressions to the points; regression line coefficients and the *R*^2^ value for the regression are shown at the panel lower right. **(C)** shows the regression-line slope coefficients averaged across ears for each frequency, for operated (red) and control (gray) ears. Error bars are 95% confidence intervals. Response recruitment varied with hearing threshold at low and mid frequencies where hearing thresholds were elevated, but not at high frequencies where hearing thresholds were within normal limits.

Comparing the data in Figures 2, 4 shows that frequencies with hearing loss (i.e., low and mid frequencies) are associated with high-slope response vs. sound-level functions, i.e. with response recruitment. In Figure 5 we explore this further. Figures 5A,B show example plots (at 0.5 and 12 kHz), of the slope metric for recruitment for each ear that had endolymphatic sac ablation, as a function of its hearing threshold at that frequency. At these two frequencies, ears with higher thresholds had higher slopes of their response growths, as would be necessary for the responses of all ears reach the same high amplitude at high sound levels. For all tested frequencies, linear-regression lines were fit to similar plots, and from each plot (one plot for each frequency and condition) we calculated linear regression coefficients. The results, in Figure 5C, show that the dependence on hearing threshold of the slope metric of response recruitment was greater at low and mid frequencies (where hearing thresholds were elevated) compared to high frequencies (where hearing thresholds were on average within normal limits). Linear-regression slope coefficients from the operated ears differed significantly (at the 0.05 level) from zero, at five of the seven low and mid frequencies (i.e., for frequencies of *0.3, 0.5*, 0.7, 1, *2, 3, and 4* kHz, with the probabilities that the slopes differed from zero being respectively: *0.014, 0.0016*, 0.174, 0.193, *0.00031, 0.000028, and 0.024*). In contrast, for high-frequencies in the operated ears, no regression slope differed from zero at the 0.05 level. In the control ears, except at the highest frequency tested, no regression slope differed from zero at the 0.05 level. Interestingly, at the lowest frequencies, the control-ear data have negative regression slopes, a pattern opposite that of the operated ears, although these negative averages were not statistically significantly different from zero. Overall, *these results suggest that clinical measurements of recruitment in patients suspected of having endolymphatic hydrops should be focused on those frequencies associated with hearing threshold elevation*.

### Histology in Ears With the Endolymphatic Sac Removed

**Figure 6:**
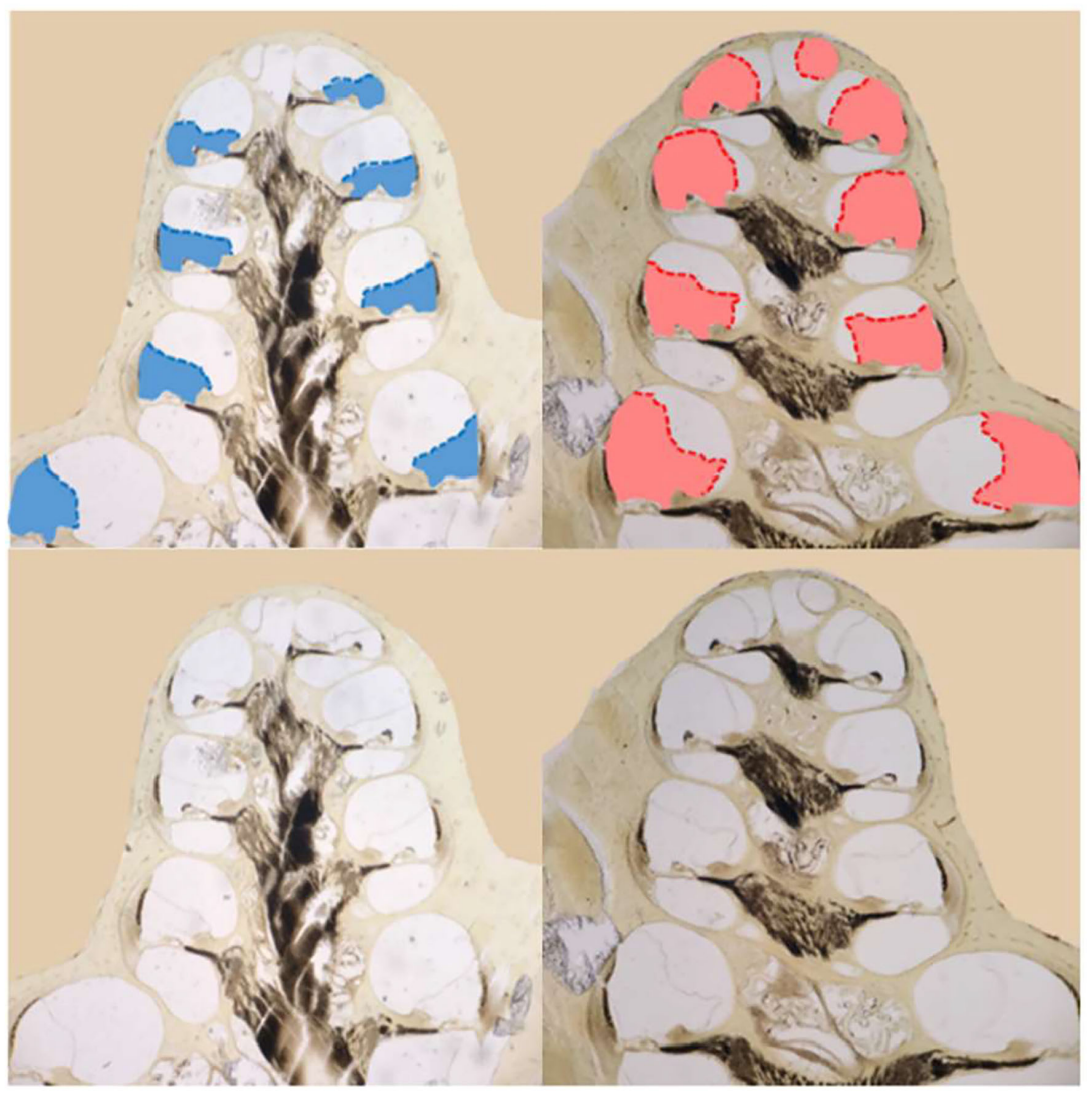
Images of mid-modiolar sections from a guinea pig that survived 30 days after a surgery to ablate the endolymphatic sac on the right side. The scala media of the right cochlea (right column) was enlarged (red) compared to that in the normal left side (left column) scala media (blue). The scala-media areas are shown in color at top and, from the same cochleae, uncolored at bottom.

Both functional and histological assessments were completed on eight ears that had the endolymphatic sac removed and on seven control ears. For each ear, we measured: (1) the average of the threshold shifts at low frequencies (≤ 1 kHz) to assess hearing loss, (2) the median at low frequencies of the slope metrics for response recruitment, and (3) the average of scala media cross sectional area in mid-modiolar sections (Figure 6) of the apical cochlear half (i.e., cochlear turns 2.5–4), to assess endolymphatic hydrops in the part of the (cochlea that has low characteristic-frequencies (20, 29).

**Figure 7:**
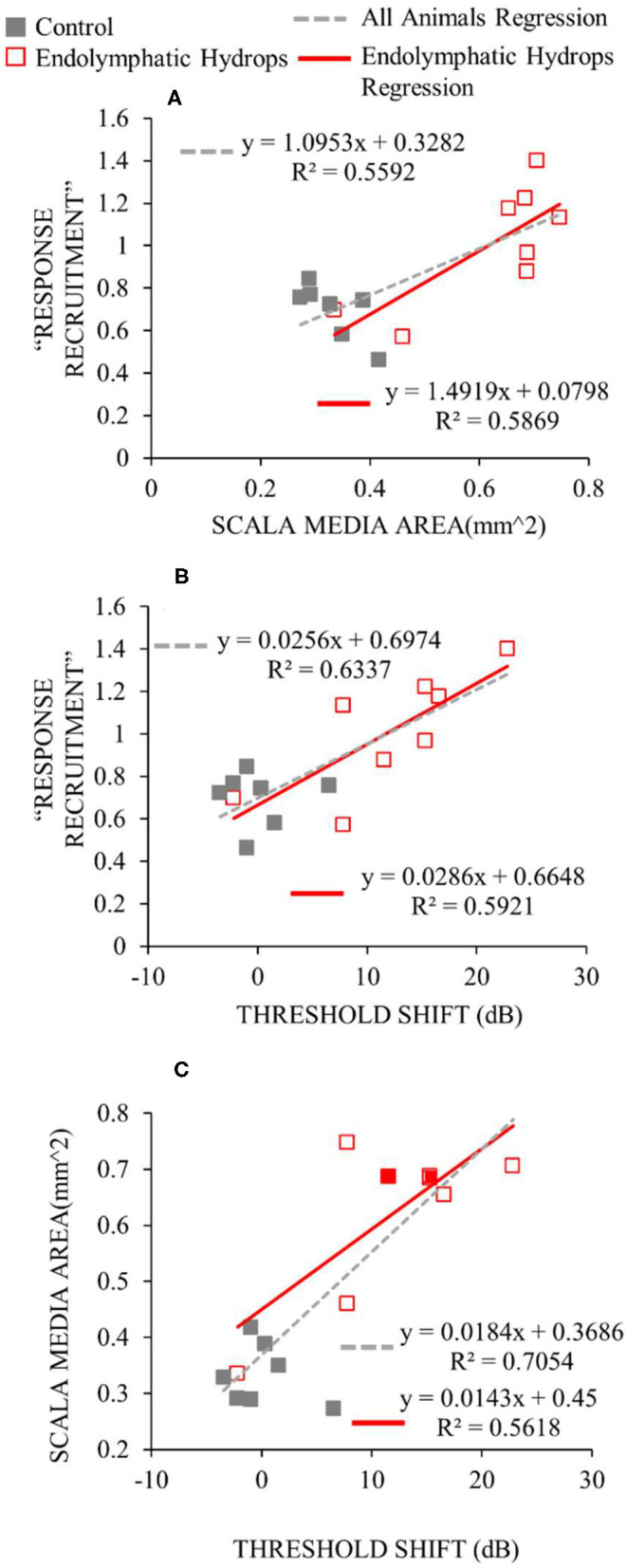
Functional and histological assessments compared. For the y-axis of **(A,B)**, the median slopes of the response growths were averaged across low frequencies (≤ 1 kHz). For the x-axis of **(A)**, the scala media area was averaged across turns in the apical cochlear half. For the x-axis of **(B,C)**, the threshold shift is the average threshold at low frequencies relative to the low-frequency average of the control ears. Data from operated ears are red and from control ears are gray. The red lines show the linear regressions to the data of the same color, while the gray dashed lines show the regressions to all the data from both control and operated ears. The linear regression parameters and the square of the Pearson's correlation coefficient are shown in each panel.

The degree of low-frequency *response* recruitment varied with the severity of apical endolymphatic hydrops (Figure 7A), consistent with the hypothesis that *loudness* recruitment varies with the severity of endolymphatic hydrops. While the correlation for operated ears hinges on two ears that had scala-media areas that were similar to unoperated ears, it was still statistically significant (*p* = 0.026). If all ears are considered, the correlation becomes highly statistically significant (*p* = 0.0014).

The degree of response recruitment varied with the extent of low-frequency hearing loss (Figure 7B), which was shown in a different way in Figure 5. Scala media area varied with low-frequency hearing threshold (Figure 7C), as was shown in Lee et al. (12), now with two additional animals (filled red symbols). Adding the two animals changed the regression very little: the regression results with the two animals removed were y = 0.014x + 0.4376; *R*^2^= 0.5731, similar to the values in Figure 7C. Overall, the Figure 7 results suggest that both *the degree of response recruitment and the low-frequency hearing loss may provide non-invasive assessments of the severity of endolymphatic hydrops*.

### Recruitment in Ears Treated With HPBCD

**Figure 8:**
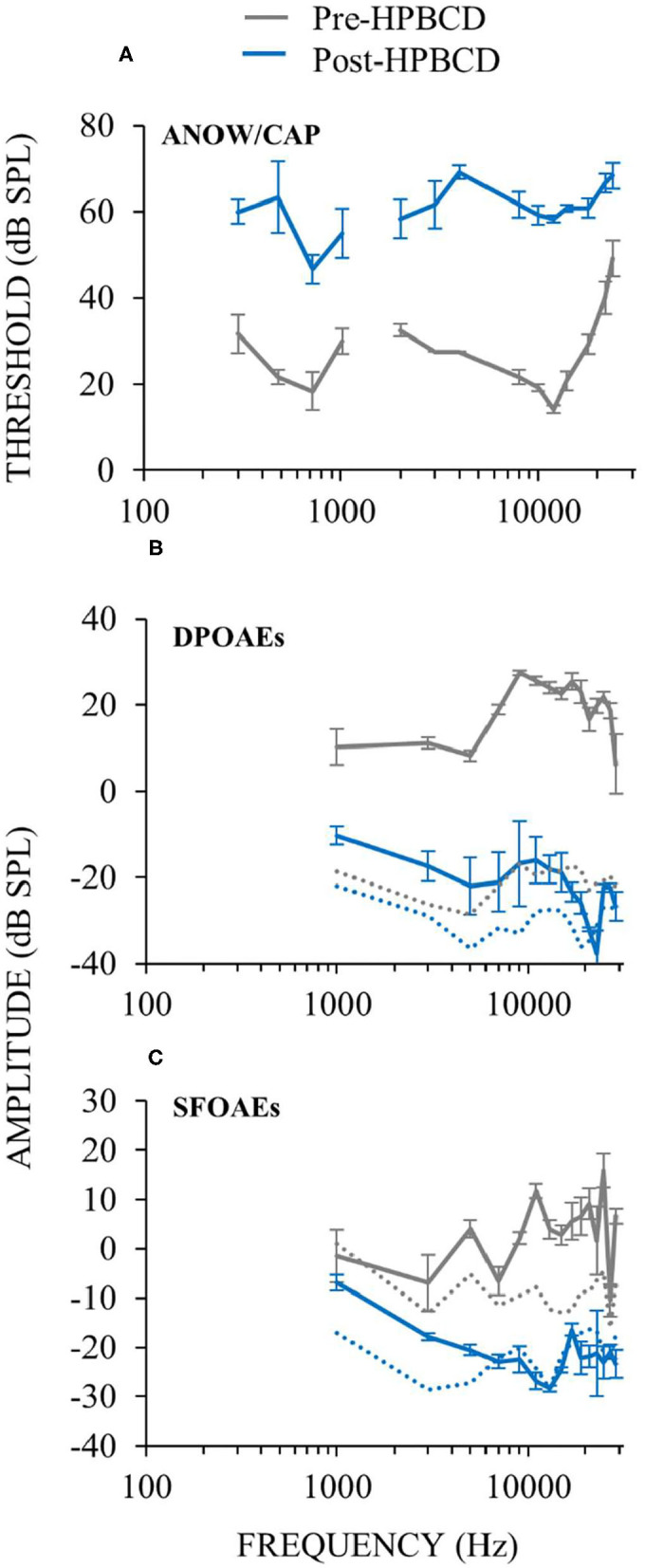
Cochlear function measurements from before (gray) and after (blue) cochlear perfusion with HPBCD in artificial perilymph. Data are averages from three animals. Error bars are one standard error of the mean. HPBCD elevated hearing thresholds **(A)**, decreased DPOAEs **(B)** and decreased SFOAEs **(C)**. OAE noise floors (dotted lines in **B,C**) for pre-HPBCD measurements were higher than post-HPBCD treatment because a paralytic was administered after the pre-HPBCD measurements.

To further explore whether auditory-nerve response recruitment originates from attenuation of cochlear amplification, we perfused HPBCD through three otherwise naïve cochleae, and measured cochlear function before and after the perfusions. HPBCD in low-doses (13 mM) has been shown to reduce OHC function while producing little or no change in IHC function or endocochlear potential (16). Auditory-nerve responses were used to quantify hearing threshold using ANOWs for low frequencies (≤ 1 kHz) and CAPs for mid (2–4 kHz) and high frequencies (8–20 kHz). Thresholds were elevated relative to control for all frequencies tested (Figure 8A), consistent with our previous HPBCD data (16). DPOAEs and SFOAEs at frequencies ≥ 1 kHz were decreased at all tested frequencies (Figures 8B,C), again consistent with our previous HPBCD data (16). Overall, the effects of acute cochlear perfusions of low-dose HPBCD were consistent with the changes being due to a reduction of cochlear amplification (30).

**Figure 9:**
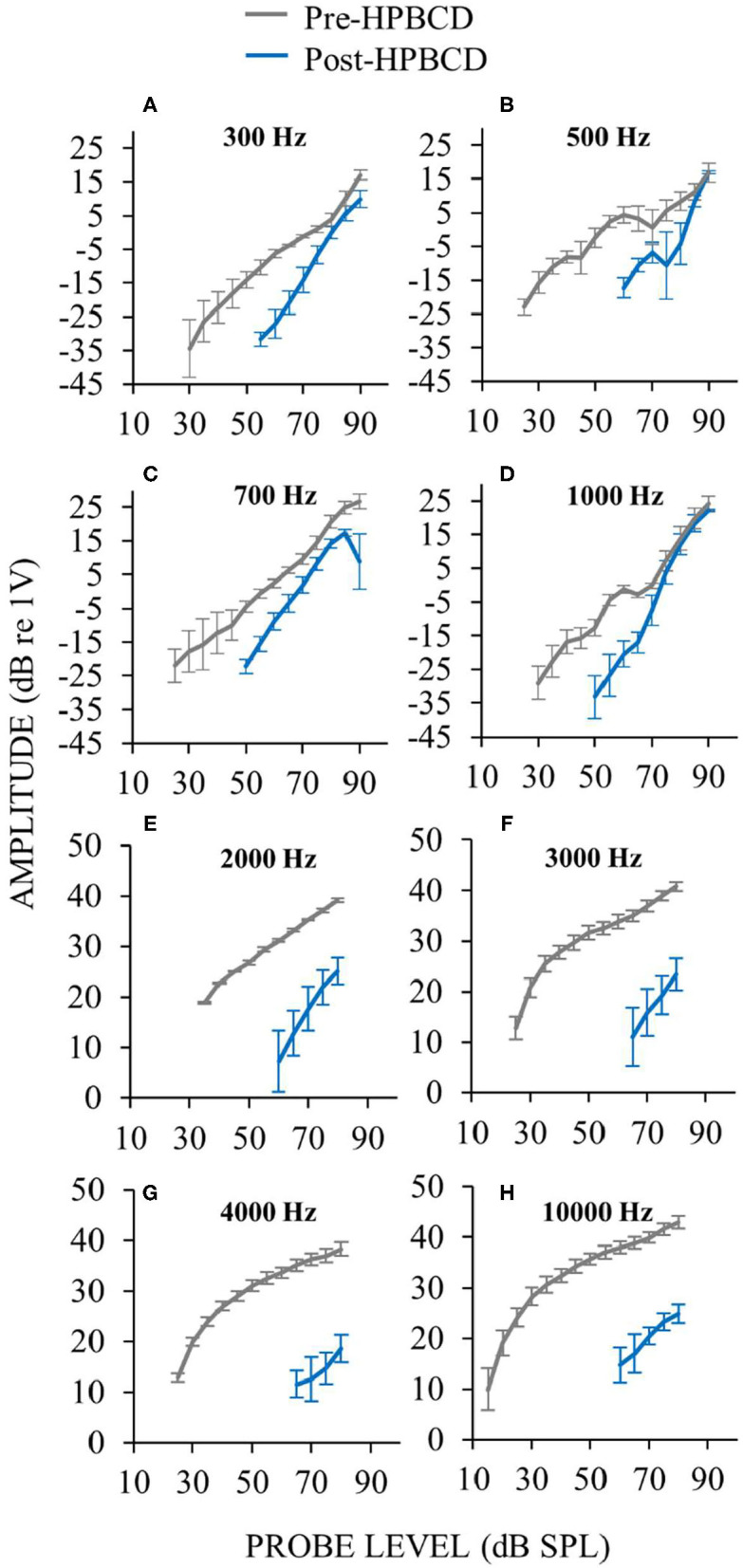
Auditory-nerve responses over a wide range of frequencies and sound level before (gray) and after (blue) cochlear perfusion with 13 mM HPBCD. ANOW was used at low frequencies 300 Hz **(A)**, 500 Hz **(B)**, 700 Hz **(C)**, 1,000 Hz **(D)**, CAPs were used for mid and high frequencies 2,000 Hz **(E)**, 3,000 Hz **(F)**, and 4,000 Hz **(G)**. Only one exemplar high frequency plot is shown (10 kHz, **H**) because plots from all high frequencies were similar. Data are averages from three ears. Error bars are one standard error of the mean. Response recruitment was seen only at low frequencies.

Auditory-nerve response amplitudes were assessed over a wide range of sound levels in the three ears perfused with HPBCD. While HPBCD elevated neural thresholds throughout the frequency range of guinea pig hearing, response recruitment was seen only at low frequencies (≤ 1 kHz). Post-perfusion thresholds were elevated but post-perfusion response amplitudes were similar to pre-perfusion amplitudes at high sound levels (Figures 9A–D). In contrast, post-perfusion measurements at mid- and high-frequencies had elevated thresholds and high-level amplitudes that were far below the range of pre-perfusion amplitudes (Figures 9E–H). Pre-perfusion responses were sometimes non-monotonic with sound level, but post-perfusion measurements were generally monotonic (Figure 9). The elevated neural thresholds and decreases in moderate-sound-level neural amplitudes in ears perfused with HPBCD are similar to our previous results (16); this is the first report of auditory-nerve responses evoked by *high sound levels* before and after HPBCD perfusion. In contrast to ears that had the endolymphatic sac removed (Figure 2), post-HPBCD-perfusion high-level response amplitudes were *not* hyper-responsive compared to control for any frequency tested (Figure 9).

### Recruitment in Operated Ears a Few Days After Endolymphatic Sac Ablation

In some animals, response recruitment was assessed a few days (i.e., 1, 2, or 4) following surgery to ablate the endolymphatic sac. Endolymphatic sac ablation produces endolymphatic hydrops that can be seen in histology at 30 postoperative days but not at a few postoperative days (12). In Lee et al. (12), we reported finding elevated hearing thresholds at low- and mid-frequencies in the first few postoperative days, and concluded that low- and mid-frequency hearing loss preceded histologically-verifiable endolymphatic hydrops. Here we address the question of whether response recruitment also precedes the development of histologically-verifiable hydrops.

**Figure 10:**
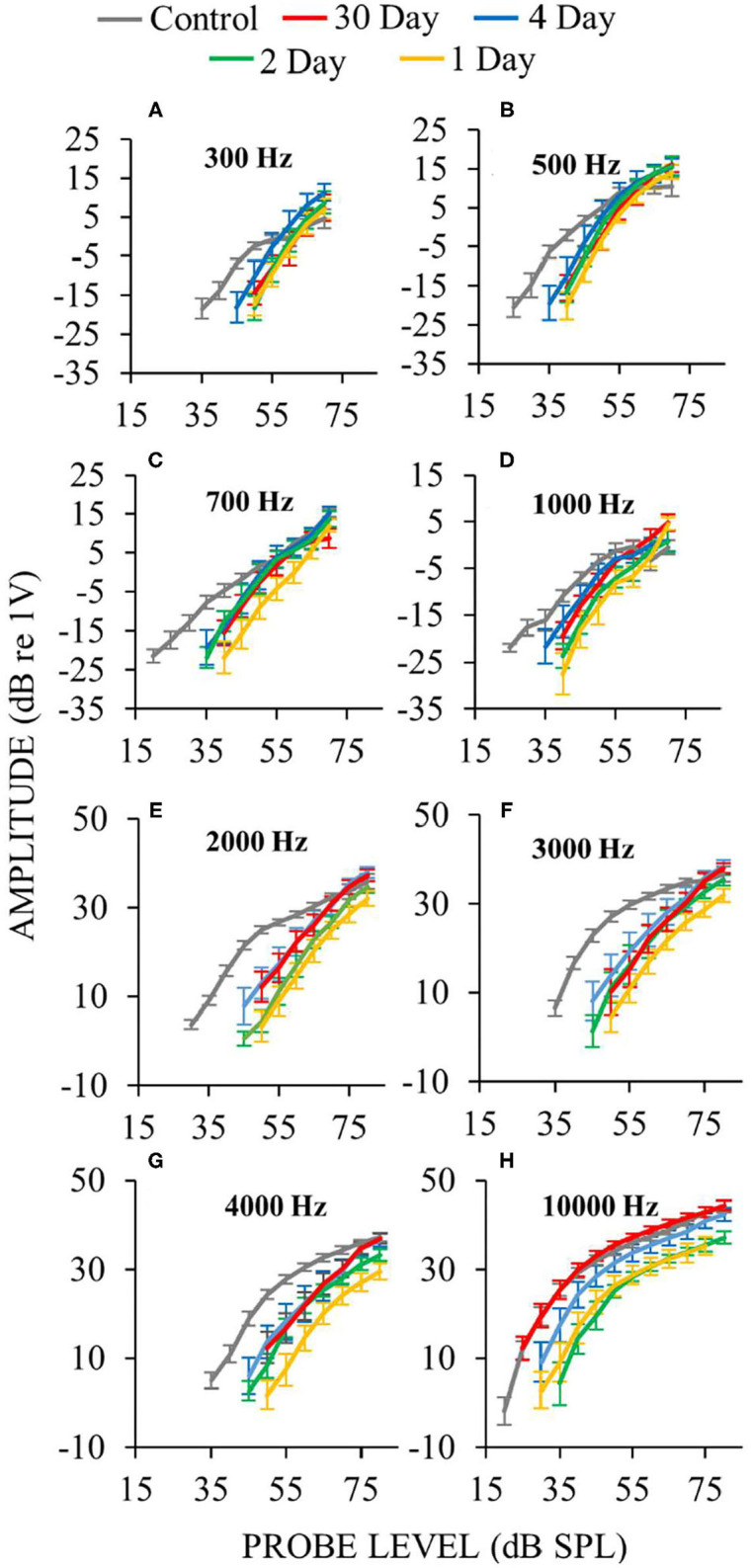
Auditory-nerve response amplitudes vs. sound-level during the first few days following endolymphatic sac surgery. Low-frequency measurements were made with ANOWs at 300 Hz **(A)**, 500 Hz **(B)**, 700 Hz **(C)**, 1,000 Hz **(D)**, mid- and high- frequency measurements were made with CAPs at 2,000 Hz **(E)**, 3,000 Hz **(F)**, 4,000 Hz **(G)**, and 10,000 Hz **(H)**. Data from eight control animals that were either naïve or underwent a sham surgery. Error bars are one standard error of the mean. The number of ears used for ANOW measurements at 30, 4, 2, and 1 postoperative days are, respectively, 17, 10, 6, and 10. The number of ears used for CAP measurements at 30, 4, 2, and 1 postoperative days are respectively 14, 10, 6, and 10. Response recruitment can be seen at low frequencies during the earliest (1–4) postoperative days before endolymphatic hydrops has been detected, indicating that response recruitment can precede the development of histologically detectable endolymphatic hydrops.

In ears assessed a few days following surgery, response recruitment at low frequencies was generally present at all postoperative days (Figure 10). In operated ears, CAP responses to high-level, low- and mid-frequency sounds were occasionally hyper-responsive compared to controls (e.g., Figure 10 at 500 Hz). In contrast, at high-frequencies response recruitment was not robustly present during the first few postoperative days. Overall these results show that endolymphatic sac ablation that causes low-frequency hearing loss and endolymphatic hydrops at 30 postoperative days, also produced response recruitment at low and mid frequencies during the first few postoperative days, even though endolymphatic hydrops cannot be seen in histology at this time.

## Discussion

### Response Recruitment Can Originate in the Cochlea

Our results show that response recruitment can have a cochlear origin and that perceptual loudness recruitment can result from attenuation of cochlear amplification. Before the results presented here, recruitment had not been convincingly shown in physiologic measurements from the auditory nerve (8, 9). Activation the medial olivocochlear efferent reflex produces recruitment in auditory-nerve CAP measurements [e.g., (31)]. However, in normal hearing, activation of medial olivocochlear efferents involves the auditory CNS, and efferent effects have not been considered as a demonstration of a cochlear origin of recruitment. The conclusion that the low-frequency response recruitment in Figures 3, 9 originated from reduced cochlear amplification rests critically on there being no dysfunction in the IHCs or their synapses with afferent auditory nerve fibers. Other reports have found hair cell and synaptic loss in ears 4–6 months after endolymphatic sac ablation (32, 33). In contrast, we have previously shown that endolymphatic hydrops does not affect IHCs, OHCs or counts of afferent synapses during the first 30 postoperative days (12, 14), which is consistent with results from human temporal bone studies of patients with Ménière's disease (22).

We assessed whether cochlear perfusions of low-dose HPBCD caused alterations of cochlear responses that are consistent with the toxicity being confined to the OHCs. Low-dose treatments of HPBCD are known to cause OHC dysfunction (16). While HPBCD elevated hearing thresholds throughout the frequency range of hearing, response recruitment occurred only at low frequencies with characteristic-frequency places in the apical half of the cochlea. It appears that acute low-dose HPBCD cochlear perfusions cause OHC-only dysfunction only in the apical cochlear half. A research question to be addressed is: do clinical patients with hearing loss from treatment with HPBCD, who had normal ears before treatment, have loudness recruitment for low-frequency sounds.

From an assessment of CAP waveforms, we found suggestive evidence that HPBCD may affect IHC function in the high-frequency basal cochlear half (16). This suggestive evidence of IHC dysfunction resulted from a 27 mM HPBCD concentration that was far higher than the 13 mM concentration used here (16). In some species, under some conditions, IHC loss has been reported following treatments with higher concentrations of HPBCD (e.g., 2,000 mg/kg delivered subcutaneously), but not with low HPBCD concentrations (34–36). In these other reports, functionally meaningful IHC loss following high-dose HPBCD administration occurred in the high-frequency basal cochlear half, not the low-frequency apical cochlear half where we found response recruitment. Another study showed that the effects of HPBCD on IHC loss were not instantaneous, but were delayed by up to 8 weeks (36). These studies also found species differences with HPBCD treatment, although they did not assess low-frequency hearing (34–36). At present, there are no reports showing that acute low-dose HPBCD affects IHC structure or function in the low-frequency apical cochlear half. Overall, the low-frequency response recruitment we found in ears acutely treated with HPBCD is adequately explained by HPBCD affecting OHC function only.

### Response Recruitment in the Auditory CNS

Following cochlear damage, measurements of CNS responses have shown that compensatory mechanism(s) in the CNS increase central-auditory gain such that responses to high-level sound can be restored to a near-normal loudness. The combination of hearing threshold elevation and central-gain increase can produce response recruitment [e.g., (37, 38)]. This response recruitment in the CNS has been interpreted as an origin perceptual loudness recruitment that is an alternative to, or in addition to, a cochlear origin (39). Measurements from the cochlear nucleus following acoustic trauma showed that, at high sound levels, neurons with “chopper” excitation properties had firing rates that were within, or sometimes greater than, normal values, which suggests that the cochlear nucleus is the most caudal origin of CNS-based changes that give rise to recruitment (40). Following destruction of IHCs or auditory nerve fibers by neurotoxic drugs, inferior colliculus field potentials were reduced less than those from the auditory nerve, which points to at least a partial recruitment mechanism between the periphery and midbrain (41–43). After almost complete ototoxic destruction of IHCs or auditory nerve fibers, measurements from the auditory cortex can be within normal limits, or even hyper-responsive, which shows adaptive restoration of responses in the auditory CNS (41, 42, 44). Together, these results suggest that the neuronal signal from a damaged cochlea is progressively amplified as it is relayed up through the CNS to the auditory cortex. The experimental manipulations used by investigators when measuring auditory-CNS responses, such as acoustic trauma and ototoxic drugs, have also been shown to be associated with or cause *behavioral* manifestations of loudness recruitment or hyperacusis (38, 45–47). In summary, an increase in central gain may be a source of loudness recruitment. We do not dispute the interpretation that recruitment can originate in the CNS. However, studies showing CNS recruitment do not rule out that recruitment can also have a cochlear origin.

### Larger-Than-Normal Responses and Hyperacusis

In ears with endolymphatic hydrops stimulated at low-frequencies and at high sound levels, we occasionally found that gross neural responses were larger than control responses (e.g., Figure 3, 500 Hz and 1 kHz, and Figure 10, 300 Hz and 500 Hz). Responses to loud sounds that are larger than normal have been interpreted as showing hyperacusis, i.e., uncomfortably loud or painful responses [e.g., (38, 41, 48)]. Compared to control responses, it is unclear exactly how much higher a response must be before accurate identification of hyperacusis is achieved. The relationship between physiologic response measurements and the perception of loudness is not completely understood, nor is the relationship between behavioral measurements and hyperacusis [e.g., (49)]. Perhaps response measurements throughout the entire auditory system need to be considered to fully assess the physiology of perceptual loudness. Measurements from the auditory efferents could also be considered in such an assessment, as they play a role in the perception of loudness, recruitment, hyperacusis, and hyper-responsiveness related to tinnitus (50, 51).

## Clinical Implications

Clinical measurement that objectively quantify loudness recruitment could be helpful to differentially diagnose endolymphatic hydrops, and to track the progression of the condition as it worsens or improves from treatment. Our data provide some guidance for how to use loudness measurements. First, ear-specific (not binaural) loudness measurements should be made, since most patients with endolymphatic hydrops are unilaterally affected, and binaural-based assessment would involve the better ear. Second, for the assessment of endolymphatic hydrops, physiologic measurements of *cochlear* recruitment may be more useful than measurements of behavioral recruitment, considering that the auditory central auditory nervous system can be a source recruitment and this could obscure cochlear recruitment [e.g., (37, 40, 41)]. It is not known how recruitment in the cochlea vs. in the CNS influences overall recruitment. Third, loudness recruitment measurements should be done only at those frequencies that have hearing threshold elevation. At frequencies not associated with elevated hearing threshold, the slope of our response amplitude-by-sound-level functions did not differ from control (Figures 3, 4). This indicates that clinicians should not test everyone with the same, or standard, frequencies because a patient might not have hearing loss (and thus not cochlear loudness recruitment) at that frequency. Forth, to identify a condition that affects the perception of loudness by attenuating cochlear amplification, OAEs alone are probably not helpful. While it has been shown that the “disparate OAE profile” (DPOAE amplitudes are reduced while SFOAE amplitudes are normal) may identify ears with endolymphatic hydrops and Ménière's disease cf. (12, 14, 52), it is not understood how OAEs from diseased ears can be used to objectively assess the perception of loudness. Fifth, cochlear microphonic measurements do not entirely originate from OHC physiologic responses (18, 53), which reduces their value for determining response recruitment. In summary, our results indicate that the combination of low-frequency hearing loss and loudness recruitment should be helpful to early identify endolymphatic hydrops and assess its severity once it develops.

Measurements of loudness recruitment should be useful in patients with Ménière's disease, considering that Ménière's patients often experience the effects of loudness recruitment. It is widely known that fitting hearing aids to patients with Ménière's disease is challenging because of their reduced dynamic range [e.g., (54, 55)]. Approaches to quantify loudness need to be developed that can be used consistently across laboratories and clinics (56). These approaches should take into account the factors outlined in the previous paragraph and the results shown in our figures.

## Data Availability Statement

The original contributions presented in the study are included in the article/supplementary material, further inquiries can be directed to the corresponding authors.

## Ethics Statement

The animal study was reviewed and approved by Washington University Institutional Animal Care and Use Committee.

## Author Contributions

JG and JL conceived experiment. SL, SG, and JL collected data. SL, RD, SG, JG, and JL interpreted results, wrote the manuscript, and approved the manuscript. JL drafted the manuscript. All authors contributed to the article and approved the submitted version.

## Funding

Research reported in this publication was supported by the National Institute of Deafness and Other Communication Disorders within the National Institutes of Health R01 DC014997 (JL).

## Conflict of Interest

The authors declare that the research was conducted in the absence of any commercial or financial relationships that could be construed as a potential conflict of interest.

## Publisher's Note

All claims expressed in this article are solely those of the authors and do not necessarily represent those of their affiliated organizations, or those of the publisher, the editors and the reviewers. Any product that may be evaluated in this article, or claim that may be made by its manufacturer, is not guaranteed or endorsed by the publisher.
